# Multiple organ dysfunction after trauma

**DOI:** 10.1002/bjs.11361

**Published:** 2019-11-06

**Authors:** E. Cole, S. Gillespie, P. Vulliamy, K. Brohi, on behalf of the Organ Dysfunction in Trauma (ORDIT) study collaborators, H. Akkad, K. Apostolidou, R. Ardley, C. Aylwin, C. Bassford, S. Bonner, A. Brooks, T. Cairns, M. Cecconi, F. Clark, G. Dempsey, E. Denison Davies, R. Docking, J. Eddlestone, D. Ellis, J. Evans, M. Galea, M. Healy, D. Horner, R. Howarth, J. Jansen, J. Jones, C. Kaye, J. Keep, D. Kerslake, J. Kilic, M. Leong, V. Martinson, B. McIldowie, S. Michael, J. Millo, M. Morgan, R. O'Leary, J. Oram, L. Ortiz‐Ruiz De Gordoa, K. Porter, S. Raby, J. Service, D. Shaw, J. D. Smith, N. Smith, M. Stotz, E. Thomas, M. Thomas, A. Vincent, G. Ward, I. Welters

**Affiliations:** ^1^ Centre for Trauma Sciences, Blizard Institute Queen Mary University of London, 4 Newark Street London E1 2AT UK

## Abstract

**Background:**

The nature of multiple organ dysfunction syndrome (MODS) after traumatic injury is evolving as resuscitation practices advance and more patients survive their injuries to reach critical care. The aim of this study was to characterize contemporary MODS subtypes in trauma critical care at a population level.

**Methods:**

Adult patients admitted to major trauma centre critical care units were enrolled in this 4‐week point‐prevalence study. MODS was defined by a daily total Sequential Organ Failure Assessment (SOFA) score of more than 5. Hierarchical clustering of SOFA scores over time was used to identify MODS subtypes.

**Results:**

Some 440 patients were enrolled, of whom 245 (55·7 per cent) developed MODS. MODS carried a high mortality rate (22·0 per cent *versus* 0·5 per cent in those without MODS; *P* < 0·001) and 24·0 per cent of deaths occurred within the first 48 h after injury. Three patterns of MODS were identified, all present on admission. Cluster 1 MODS resolved early with a median time to recovery of 4 days and a mortality rate of 14·4 per cent. Cluster 2 had a delayed recovery (median 13 days) and a mortality rate of 35 per cent. Cluster 3 had a prolonged recovery (median 25 days) and high associated mortality rate of 46 per cent. Multivariable analysis revealed distinct clinical associations for each form of MODS; 24‐hour crystalloid administration was associated strongly with cluster 1 (*P* = 0·009), traumatic brain injury with cluster 2 (*P* = 0·002) and admission shock severity with cluster 3 (*P* = 0·003).

**Conclusion:**

Contemporary MODS has at least three distinct types based on patterns of severity and recovery. Further characterization of MODS subtypes and their underlying pathophysiology may lead to future opportunities for early stratification and targeted interventions.

## Graphical abstract

As trauma practice advances, postinjury multiple organ dysfunction syndrome (MODS) is changing. The aim of this study was to characterize contemporary MODS in trauma critical care. Three patterns of MODS were identified, all present on admission, with distinct clinical associations. SOFA, Sequential Organ Failure Assessment.

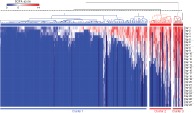

Present from admission with subtypes having different patterns of recovery

## Abstract (es)

**Antecedentes:**

La naturaleza del síndrome de disfunción orgánica múltiple (*Multiple Organ Dysfunction Syndrome*, MODS) resultante de un traumatismo está evolucionando a medida que avanzan las prácticas de reanimación y más pacientes sobrevive a las lesiones y pueden recibir cuidados críticos. El objetivo de este estudio fue caracterizar los subtipos actuales MODS en atención crítica de trauma a nivel poblacional.

**Métodos:**

Los pacientes adultos ingresados en unidades de cuidados intensivos de trauma se incluyeron en este estudio de prevalencia puntual de 4 semanas. MODS se definió como una puntuación total diaria de la escala de Evaluación de Fallo Orgánico Secuencial (*Sequential Organ Failure Assessment*, SOFA) > 5. Se utilizó el agrupamiento jerárquico de las puntuaciones SOFA a lo largo del tiempo para determinar los subtipos MODS.

**Resultados:**

Se incluyeron 440 pacientes, de los cuales 245 (56%) presentaron MODS. MODS conllevó una alta mortalidad (22% *versus* 1%, *P* < 0,001) y 24% de las muertes fueron precoces, durante las primeras 48 horas tras el traumatismo. Se identificaron tres patrones de MODS, estando todos presentes al ingreso. En el tipo 1, MODS se resolvió de forma temprana, con una mediana de tiempo de recuperación de 4 días y una mortalidad del 14%. El tipo 2 presentaba un tiempo de recuperación retardado (mediana 13 días) y una mortalidad del 35%. El tipo 3 presentaba un tiempo de recuperación prolongado (mediana 25 días) y una mortalidad asociada alta del 46%. El análisis multivariable reveló asociaciones clínicas diferentes para cada tipo de MODS, con la administración de cristaloides durante 24 horas fuertemente asociada al tipo 1 (*P* < 0,001); el traumatismo craneal al tipo 2 (*P* < 0,01); y la gravedad del shock al ingreso al tipo 3 (*P* < 0,01).

**Conclusión:**

Los MODS actuales presentan al menos tres tipos distintos basados en patrones de gravedad y recuperación. La caracterización de los subtipos de MODS y su fisiopatología subyacente puede contribuir a futuras oportunidades de estratificación temprana e intervenciones dirigidas.

## Introduction

Multiple organ dysfunction syndrome (MODS) is common in critically injured patients who survive the initial insult, and is associated with poor outcomes^1^. MODS is responsible for a large proportion of the healthcare resources associated with acute trauma care^2^. As early‐phase management strategies such as damage control resuscitation have been introduced and more injured patients are surviving to reach critical care, the nature of MODS appears to be changing^3^. Reports of differences in patterns of evolution, severity and outcome are challenging the previously accepted clinical concepts of ‘early and late onset’ or bimodal peaks of MODS^4^, ^5^, ^6^, ^7^, ^8^, ^9^. Determining the incidence, pattern and outcomes of MODS in the modern era may help to identify therapeutic opportunities and inform study design for future research.

With more early survivors of severe injury, MODS remains a determinant of poor outcomes and a continued challenge for trauma systems^2^, ^7^. The pathophysiology of MODS is a subject of debate in the literature. Some of the uncertainty may be due to the existence of discrete forms of MODS with unique pathophysiologies. Recent studies^4^, ^10^, ^11^ have described protracted forms of MODS associated with ageing, immunosuppression, infection and catabolism. The national incidence of these contemporary MODS subtypes, their resource use and associated outcomes are not known. Characterizing these different MODS subtypes is important if progress is to be made in understanding their underlying pathophysiologies, in developing new diagnostic and therapeutic approaches, and for the design of clinical studies to test them.

The overall aim of this study was to characterize contemporary MODS subtypes in trauma critical care at a population level. The primary aim was to establish the overall incidence of MODS, and to describe patterns of severity and recovery, and their associated outcomes. Subsequent aims were to examine different patterns of MODS onset and recovery, and describe admission characteristics associated with any MODS subtypes identified.

## Methods

England, Wales and Scotland have an organized system of regional trauma networks, each with a major trauma centre (MTC) (level I equivalent) designated to manage the most severely injured patients in a geographical region. In the UK, MTCs should adhere to trauma resuscitation guidelines from the National Institute for Health and Care Excellence^12^. All MTCs were invited to participate, and injured adult patients (aged at least 16 years) requiring admission to a critical care unit between 00·01 hours on 1 June and 23·59 hours on 30 June 2016 were eligible for inclusion. Study approval was provided by the National Health Service Research Ethics Committee (REC), Health Research Authority and Scotland REC A (reference 15/SS/0170). Patients were enrolled in the study after admission to critical care, and informed consent was obtained from the patient or a consultee.

### Study procedures

Procedures and training were provided via the Organ Dysfunction in Trauma (ORDIT) study webpages^13^. Data on demographic characteristics, injury severity, admission physiology and resuscitation in the first 24 h were recorded. Patients were reviewed daily in critical care until discharge or death.

### Definitions

The presence and evolution of MODS was determined based on Sequential Organ Failure Assessment (SOFA) scoring^14^, ^15^. SOFA was chosen as it is used widely in critical care internationally. It has been validated for use in injured patients, including its application from the day of admission^16^, and has a good balance of sensitivity and specificity in predicting unfavourable outcome after severe injury^14^. SOFA scores were measured daily from admission as described in earlier critical care studies^16^, ^17^, ^18^. MODS was defined by the occurrence of a total SOFA score greater than 5, affecting two or more organs^1^, ^4^, ^19^. Recovery was deemed to have occurred when the SOFA score fell and remained below 6. Traumatic brain injury (TBI) was diagnosed when the head Abbreviated Injury Score was 3 or higher. Secondary outcomes were in‐hospital mortality, time spent on ventilator, and duration of critical care and total hospital stay.

### Statistical analysis

Categorical variables were analysed using the χ^2^ or Fisher's exact test. Continuous data had a non‐normal distribution according to the Shapiro–Wilk test, and were evaluated by means of non‐parametric Mann–Whitney *U* and Kruskal–Wallis tests. Multiple comparisons of individual pairs were done with Bonferroni corrections. Unsupervised hierarchical clustering was performed to define MODS cohorts using Morpheus online software (Broad Institute, Massachusetts, USA). Previous clinical studies^18^, ^20^, ^21^ have used hierarchical clustering analysis to classify patients within a population into discrete clusters or phenotypes on the basis of clinical data. Raw total SOFA score data for each patient on each of days 1–28 (or day of discharge from critical care or death, if earlier) were entered into the data matrix. A SOFA score of 0 was assigned on discharge from the ICU (unless the patient was readmitted). For patients who died during the 28‐day interval, no score was allocated after the date of death. These data were treated as missing in the clustering analysis and were excluded from all computations involving the rows within which they occurred. Heatmaps were generated using the Euclidean distance between observations and complete linkage between clusters. Complete linkage has been reported to produce more meaningful separation between clusters than average linkage^22^, ^23^ and standard Grubbs' test was used to identify outliers^24^. The number of clusters was chosen subjectively by selecting a single threshold height that maintained a reasonable subcluster sample size. The sensitivity and specificity of the clusters' ability to detect MODS on each day was used to determine serial Youden indices and reported as sensitivity + specificity − 1.

Multivariable logistic regression models were used to describe the association between admission or treatment variables and MODS. Variables with *P* < 0·100 in univariable testing were entered into the regression analysis. Results are presented as odds ratios (ORs) with 95 per cent confidence intervals. The calibration and goodness of fit of the logistic regression models were evaluated using the χ^2^ Hosmer–Lemeshow (HL) test, and model discrimination was assessed by means of area under the receiver operating characteristic curve (AUROC). Correlations between crystalloid use and MODS were described using Spearman's rank correlation coefficients.

Data analysis was conducted using SPSS® version 21 (IBM, Armonk, New York, USA).

## Results

All 29 adult major trauma centres in England, Wales and Scotland participated in the study. In total, 446 patients were admitted to critical care during the 1‐month period, of whom six died within the first 24 h after injury. Of the remaining 440 patients, 245 (55·7 per cent) developed MODS (*Table* 1). The onset of MODS was on the day of admission in the majority of patients (94·3 per cent). The remainder developed MODS on day 2 (5·3 per cent) or day 3 (0·4 per cent). Only two patients experienced a second episode of MODS, one on day 7 and one on day 11. The mean admission SOFA score was 8·5 (95 per cent c.i. 8·2 to 8·8), and severity peaked on day 2 with a score of 8·8 (8·5 to 9·1). Respiratory and cardiovascular dysfunction were the greatest contributors to MODS (97·1 per cent and 91·0 per cent of patients), followed by central nervous system (CNS) dysfunction (88·1 per cent). Coagulation dysfunction affected 57·5 per cent; liver and renal systems had the lowest incidence of dysfunction (36·7 and 26·7 per cent respectively). Organ dysfunction patterns were similar in patients with and without TBI (*Table* *S1*, *Figs* *S1* and *S2*, supporting information).

**Table 1:** Admission characteristics, injuries and outcomes

	Cluster 1 No MODS (*n* = 195)	Cluster 1 MODS (*n* = 167)	Cluster 2 MODS (*n* = 54)	Cluster 3 MODS (*n* = 24)	All MODS (*n* = 245)
**Admission variables**					
Age (years)*	48 (29–63)	47 (29–68)	47 (28–60)	42 (28–57)	47 (29–65)
Sex ratio (M : F)	140 : 55	122 : 45	43 : 11	20 : 4	185 : 60
Blunt injury	166 (85·1)	151 (90·4)	51 (94)	20 (83)	222 (90·6)
First GCS score*	14 (9–15)	14 (6–15)	12 (4–15)	14 (9–15)	13 (6–15)†
First systolic BP measurement (mmHg)*	127 (107–141)	125 (103–145)	125 (110–143)	128 (110–144)	125 (104–145)
< 90	11 (5·6)	19 (11·4)	2 (4)	2 (8)	23 (9·4)
First base deficit measurement (mEq/l)*	0 (–1·2 to 1·1)	0 (–1·1 to 5·1)	0 (–0·2 to 5·0)	7·5 (0·3 to 11·1)	0·1 (0·4–6·7)†
Crystalloid (l per 24 h)*	1·2 (1·0–2·0)	3·0 (2·0–4·5)	2·3 (1·7–3·8)	3·7 (2·2–5·0)	2·9 (2·0–4·5)†
RBC (units per 24 h)*	3 (2–4)	4 (2–6)	4 (2–7)	4 (2–11)	4 (2–7)†
FFP (units per 24 h)*	3 (2–4)	4 (2–8)	4 (2–6)	4 (2–8)	4 (2–8)†
FFP : RBC ratio*	0·1 (0–0·6)	0·6 (0–1·0)	0·5 (0–1·0)	0·5 (0–0·8)	0·5 (0–1·0)
Traumatic brain injury	31 (15·9)	56 (33·5)	34 (63)	9 (38)	99 (40·4)†
Injury Severity Score*	17 (9–26)	25 (16–33)	29 (25–43)	31 (19–45)	25 (18–36)†
APACHE II score*	9 (6–13)	14 (10–19)	15 (10–22)	18 (11–24)	14 (10–20)
**Outcomes**					
Death	1 (0·5)	24 (14·4)	19 (35)	11 (46)	54 (22·0)
Time on ventilator (days)*	1 (1–2)	3 (1–5)	11 (10–15)	16 (12–24)	4 (2–11)
Duration of critical care stay (days)*	3 (2–5)	7 (4–14)	20 (16–27)	36 (32–47)	11 (5–19)
Total duration of hospital care (days)*	9 (5–20)	22 (12–33)	39 (25–62)	53 (39–104)	26 (13–40)

Values in parentheses are percentages unless indicated otherwise;

* values are median (i.q.r.). Only 21 patients (4·8 per cent) received colloids; median amount in all groups was 0 (0–0) l per 24 h. MODS, multiple organ dysfunction syndrome; GCS, Glasgow Coma Scale; RBC, red blood cells; FFP, fresh frozen plasma; APACHE II, Acute Physiology And Chronic Health Evaluation II.

† Variables with *P* < 0·100 in univariable analysis of all MODS group *versus* no MODS, or comparisons between clusters 1–3 (χ^2^ or Fisher's exact test for categorical variables; Mann–Whitney *U* test for continuous variables) were entered into multivariable models.

Some 22·0 per cent of patients with MODS died, compared with 0·5 per cent of the group without MODS (*Table* 1). Among the 310 patients (70·5 per cent) without TBI, the mortality rate in those with MODS was 15·1 per cent, compared with 0·6 per cent in those without MODS (*Table* *S1*, supporting information). The overall median time to death in patients with MODS was 6 (i.q.r. 2–10) days and 24·0 per cent of all MODS deaths occurred within the first 48 h. For survivors, the median time with MODS was 10 (i.q.r. 5–17) days. In total, the 245 patients with MODS used 2656 critical care bed‐days and spent 5872 days in hospital.

**Figure 1:**
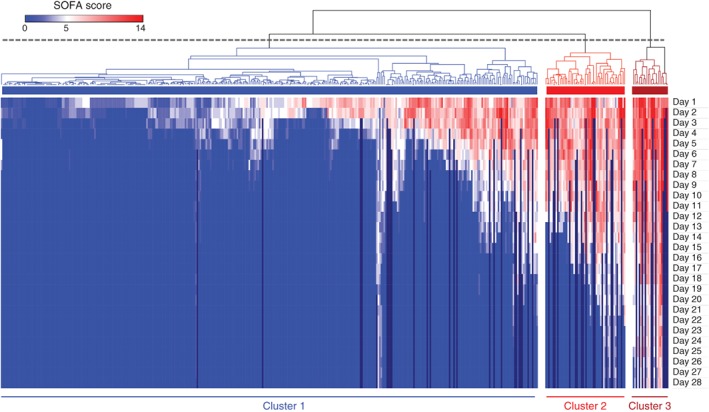
Unsupervised hierarchical clustering of sequential organ failure assessment scores from admission to day 28 Shades of red within the heatmap indicate patients with multiple organ dysfunction (MODS), defined by a Sequential Organ Failure Assessment (SOFA) score of 6 or higher. Patients discharged from critical care were assigned a score of 0 (blue). Patients who died within 28 days had missing scores after the date of death (black). Dashed line indicates the level of dendrogram transection used for analysis.

**Figure 2:**
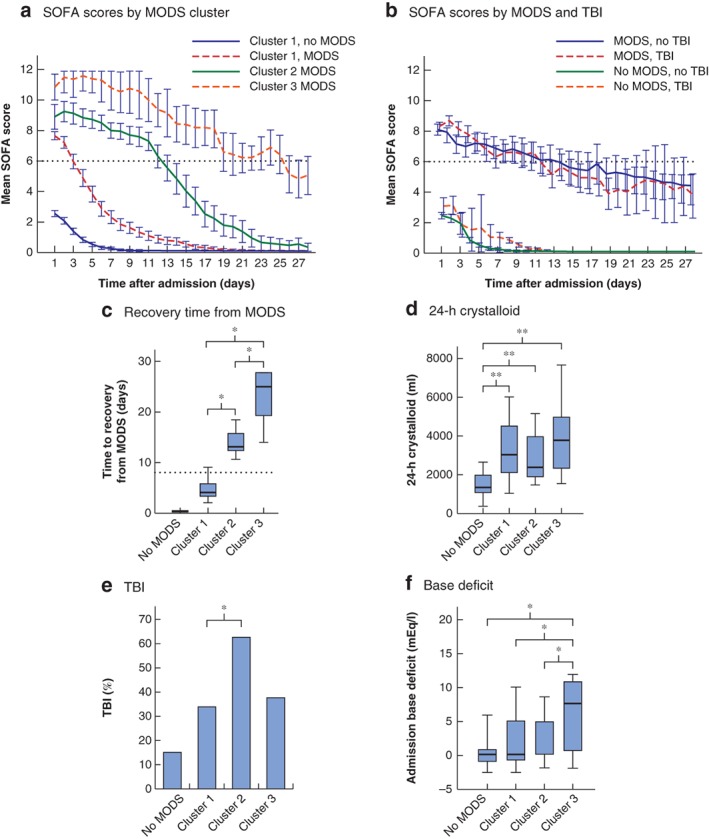
Multiple organ dysfunction syndrome development, recovery and associations Mean Sequential Organ Failure Assessment (SOFA) score in relation to time after admission to hospital by **a** multiple organ dysfunction syndrome (MODS) clusters and **b** presence of MODs and traumatic brain injury (TBI); error bars represent 95 per cent confidence intervals and the dotted line indicates the threshold for diagnosing MODS. **c** Time to resolution of MODS in survivors, defined as time after admission to first reaching a SOFA score of less than 6; the dotted line represents day 8 (discrimination between MODS status in cluster 1 and that in clusters 2 and 3). **d** Crystalloid administration over 24 h. **e** Percentage of patients with TBI (head Abbreviated Injury Score of at least 3). **f** Admission base deficit per cluster. Median values (bold line), i.q.r. (box) and error bars (range) are shown in **c**,**d** and **f**. *P < 0·010 (**c**,**d**,**f** Kruskal–Wallis test with multiple comparisons of selected cluster groups using Bonferroni correction; **e** χ^2^ test).

Hierarchical clustering analysis of daily SOFA scores identified three high‐level clusters of different patient recovery patterns (*Fig*. 1). Cluster 1 was the largest with 362 patients, of whom 167 developed MODS (68·2 per cent of all patients with MODS). All 54 patients in cluster 2 developed MODS (22·0 per cent), as did the 24 patients in cluster 3 (9·8 per cent). Admission SOFA scores for patients with MODS were higher in clusters 2 and 3: mean SOFA score on day 1: 7·7 in cluster 1 *versus* 8·9 in cluster 2 (*P* = 0·002) and 10·9 in cluster 3 (*P* < 0·001). SOFA scores for patients with MODS in cluster 1 began improving immediately, and in survivors had fallen below MODS thresholds by day 5. SOFA scores initially worsened for patients in clusters 2 and 3 before beginning to resolve from day 3; they did not fall below admission levels until day 5 (*Fig*. 2 *a*). Patterns of recovery did not appear to be affected by the presence of TBI (*Fig*. 2 *b*). MODS took longer to resolve in patients in cluster 2 (median 13 (i.q.r.12–16) days), whereas those in cluster 3 had a very protracted duration of organ dysfunction (25 (19–28) days) (*Fig*. 2 *c*).

MODS had resolved in 71·9 per cent of patients (120 of 167) in cluster 1 by day 7, and no patient in this cluster still had MODS after 11 days. Patients with MODS in cluster 1 were best discriminated from those in clusters 2 and 3 by their MODS status on day 8. Some 88 per cent of patients still with MODS on day 8 were in clusters 2 or 3 (sensitivity 99 per cent, specificity 93 per cent for being in clusters 2/3, Youden index 0·91). Day 18 was the best discriminator between patients in clusters 2 and 3; 79 per cent of patients who still had MODS on day 18 were in cluster 3 (sensitivity 85 per cent, specificity 94 per cent for being in cluster 3, Youden index 0·78).

The clusters also had different outcome profiles. Patients with MODS in cluster 1 had a mortality rate of 14·4 per cent, rising to 35 per cent in cluster 2 (*P* < 0·001) and 46 per cent in cluster 3 (*P* < 0·001) (*Table* 1). The duration of critical care stay for patients in cluster 3 was double that of those in cluster 2 (36 and 20 days respectively; *P* < 0·001) and hospital stay was almost 40 per cent longer (53 *versus* 39 days; *P* = 0·030) (*Table* 1).

Admission characteristics associated with the development of MODS in each cluster were analysed. MODS cluster 1 was the only cluster in which the development of MODS was not associated with injury severity (*Table* 2), despite high Injury Severity Scores (median 25) (*Table* 1). The cluster 1 MODS group was most strongly associated with the volume of crystalloid administered within the first 24 h (OR 1·32) (*Table* 2 and *Fig*. 2 *d*). Cluster 2 contained the greatest proportion of patients with TBI (63 per cent) (*Fig*. 2 *e*) and brain injury was strongly associated with this cluster (OR 3·51) (*Table* 2). Cluster 3 was the only MODS subtype with patients shocked on arrival (median base deficit 7·5 *versus* 0 mEq/l in all other MODS clusters; *P* = 0·004) (*Fig*. 2 *f*). Both admission base deficit and 24‐h crystalloid use were independently associated with the development of prolonged MODS in cluster 3 (OR 1·11, *P* = 0·003, and OR 1·24, *P* = 0·019 respectively) (*Table* 2).

**Table 2:** Multivariable logistic regression analysis of factors associated with the development of multiple organ dysfunction syndrome

	Cluster 1 MODS (*n* = 167)		Cluster 2 MODS (*n* = 54)		Cluster 3 MODS (*n* = 24)		All MODS (*n* = 245)	
	Odds ratio	*P*	Odds ratio	*P*	Odds ratio	*P*	Odds ratio	*P*
First GCS score	1·02 (0·91, 1·09)	0·960	0·93 (0·85, 1·01)	0·091	–		1·06 (0·92, 1·20)	0·374
First base deficit measurement	1·02 (0·96, 1·09)	0·424	0·99 (0·92, 1·07)	0·851	1·11 (1·01, 1·21)	0·003	1·04 (0·94, 1·16)	0·408
Crystalloid (l per 24 h)	1·32 (1·07, 1·63)	0·009	0·99 (0·81, 1·21)	0·928	1·24 (1·03, 1·51)	0·019	3·09 (1·76, 5·43)	< 0·001
RBC (units per 24 h)	1·02 (0·91, 1·13)	0·705	–		–		1·22 (0·98, 1·51)	0·069
FFP (units per 24 h)	1·12 (0·86, 1·45)	0·370	–		–		1·16 (0·73, 1·83)	0·516
Traumatic brain injury	2·00 (0·66, 6·10)	0·474	3·5 (1·57, 7·82)	0·002	0·98 (0·35, 2·06)	0·973	4·29 (0·39, 46·80)	0·232
Injury Severity Score	1·01 (0·93, 1·03)	0·513	1·03 (1·00, 1·06)	0·015	1·03 (1·00, 1·06)	0·036	1·06 (1·01, 1·10)	0·007

Values in parentheses are 95 per cent confidence intervals. Variables with *P* < 0·100 in univariable analysis of all multiple organ dysfunction syndrome (MODS) group *versus* no MODS, or comparisons between clusters 1–3 were entered into multivariable models. GCS, Glasgow Coma Scale; RBC, red blood cells; FFP, fresh frozen plasma. Cluster 1 model: area under receiver operating characteristic curve (AUROC) 0·79 (95 per cent c.i. 0·61 to 0·88), Hosmer–Lemeshow (HL) χ^2^ = 5·8, *P* = 0·664; cluster 2 model: AUROC 0·73 (0·63 to 0·82), HL χ^2^ = 4·4, *P* = 0·811; cluster 3 model: AUROC 0·80 (0·72 to 0·88), HL χ^2^ = 7·0, *P* = 0·535; all MODS model: AUROC 0·91 (0·85 to 0·97), HL χ^2^ = 8·9, *P* = 0·347.

**Figure 3:**
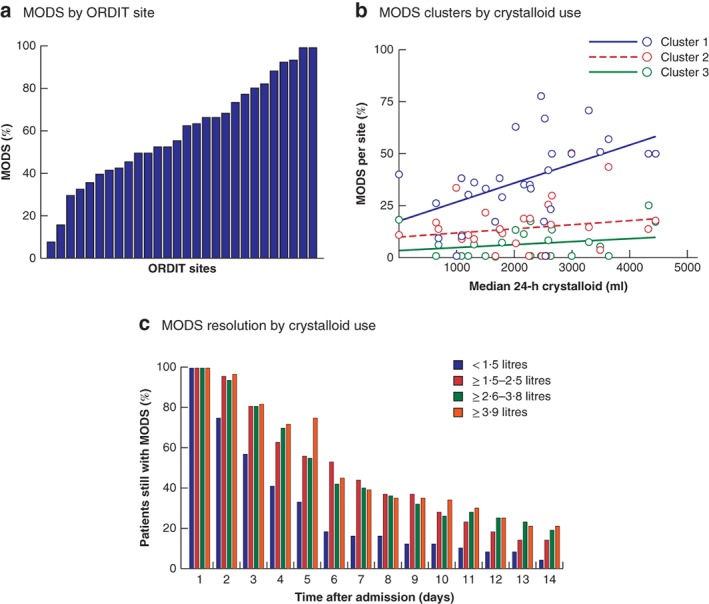
Multiple organ dysfunction syndrome by site and crystalloid use **a** Percentage of patients who developed multiple organ dysfunction syndrome (MODS) by Organ Dysfunction in Trauma (ORDIT) site in rank order. **b** Scatter plot showing percentage of patients per site with MODS in clusters 1, 2 and 3 in relation to median crystalloid use per site (cluster 1 MODS: *r*_s_ = 0·65, *P* < 0·001; cluster 2 MODS: *r*_s_ = 0·15, *P* = 0·424; cluster 3 MODS: *r*_s_ = 0·56, *P* = 0·001; all MODS: *r*_s_ = 0·66, *P* < 0·001). **c** Percentage of patients remaining in MODS on each day after admission in quartiles of initial 24‐h crystalloid administration.

The median volume of crystalloid administered in patients with MODS varied across sites from 1 to 5·2 litres, whereas the incidence of MODS ranged from 8 to 100 per cent (*Fig*. 3 *a*; *Table* *S2*, supporting information). There was a strong correlation between crystalloid use at individual sites and the development of MODS in cluster 1 (*r*_s_ = 0·66, *P* < 0·001). A modest correlation was seen in cluster 3 (*r*_s_ = 0·56, *P* = 0·001), but not in cluster 2 (*r*_s_ = 0·15, *P* = 0·424) (*Fig*. 3 *b*). Overall, MODS resolved in a shorter time among patients who received less than 1·5 litres of crystalloid in the first 24 h than in those receiving greater volumes (*Fig*. 3 *c*). By day 8 after injury almost twice the proportion of patients who had received more than 1·5 litres of crystalloid still had MODS compared with those who had received 1·5 litres or less (36·0 *versus* 19·0 per cent; *P* = 0·010). The median duration of critical care stay was longer for patients with MODS who received more than 1·5 litres of crystalloid in the first 24 h (7 *versus* 5 days; *P* < 0·001) (*Table* *S3*, supporting information).

## Discussion

This was a national point‐prevalence study of MODS after traumatic injury in the era of contemporary damage control resuscitation, neurocritical care and regional trauma systems. MODS was common in this critical care population and had a high associated mortality rate and resource use. A total of 7604 injured patients were admitted to critical care units in England and Wales in 2016 (Trauma Audit and Research Network, personal communication). It would be predicted nationally to be over 3300 injured patients in critical care with MODS annually, requiring 33 000 critical care bed‐days, of whom 750 will die. Survivors will require 63 750 days in hospital overall, and may suffer long‐term impairment to physical and psychological outcomes^25^.

Overall outcomes appear to have improved for patients with MODS in comparison to those described in previous studies. The mortality rate of 22·0 per cent among patients with MODS in the present study compares with 33–36 per cent in a study from the Glue Grant consortium in the USA, which examined temporal trends in patients with MODS between 2003 and 2010^2^. In the present study there was a preponderance of cardiovascular dysfunction in the initial days after injury. Given that 24·0 per cent of deaths occurred in this time frame, it may be that cardiovascular dysfunction is the major component of MODS in the damage control resuscitation era.

MODS appeared to be present in injured patients on the first day of admission and had at least three distinct recovery patterns: an early resolving form and two persistent types. In cluster 1, MODS had resolved in 31 patients (7·0 per cent of 440 study patients) within the first 48 h, and this group would not have been included in other studies that defined MODS as occurring after day 2. In contrast, nearly one‐third of patients with MODS in this study had a prolonged form (cluster 2 or 3) taking a median of 15 days to resolve. Historically, MODS has had a bimodal distribution, with a second peak in incidence occurring between 7 and 14 days after injury^26^. This second peak has been declining^9^ and appears to have all but disappeared in contemporary MODS, with only three patients in the present study developing MODS after day 3. Contemporary MODS is characterized by respiratory, cardiovascular and haemostatic dysfunction. Respiratory and cardiovascular dysfunction were nearly universal in these patients with MODS, whereas older studies^2^ have described involvement of these organ systems at around 50 per cent. Patients with different recovery trajectories were indistinguishable on admission solely by examination of total or component SOFA scores, but there were differences in injury patterns, shock severity and early management associated with the subsequent development of the MODS subtypes.

Crystalloid use in the first 24 h after injury was the only clinical variable associated with the cluster 1 early resolving MODS subtype. Crystalloids are known to have proinflammatory effects on coagulation and the endothelium^27^, ^28^, ^29^, and volume administered is associated with increased organ injury such as acute respiratory distress syndrome and abdominal compartment syndrome. Previous trauma studies^30^, ^31^ reported a link between organ dysfunction and administration of large volumes of crystalloid in the first 12 or 24 h after injury. Current national trauma haemorrhage guidance^32^ reflects this, recommending that crystalloids are avoided during damage control resuscitation. It is likely therefore that the majority of crystalloid administration occurs in the critical care unit. Only fluid use in the first 24 h was examined, but this volume replacement may persist for some days.. Further research is needed to explore causality in this relationship and identify optimal strategies for ongoing volume replacement in these patients.

TBI characterized the group of patients whose MODS tended to persist, with a delayed recovery between days 8 and 18. Brain injury is thought to potentially confound the assessment of MODS and its resolution^14^, ^33^. In the present study, there was little to differentiate patients with and without MODS in terms of severity or pattern of TBI. However, patients with TBI and MODS appear to have a specific, separate clinical trajectory, not limited to the CNS component of the SOFA score alone. Further investigation is required to determine whether this is due to extracranial effects of TBI or current management paradigms. For example, endogenous or exogenous catecholamines have both been postulated as responsible for some of the observed effects^34^, ^35^, ^36^. The presence of MODS is known to worsen outcomes for those with severe TBI^37^, ^38^, so research focusing on this specific group of patients may elucidate underlying mechanisms and lead to real improvements for patients with brain injury.

Patients in cluster 3 with the prolonged form of MODS were the smallest cohort, but had the worst outcomes and consumed a disproportionate amount of critical care resources. Previous studies^39^, ^40^ of indolent immunosuppressive dysfunction have focused on elderly patients, but there was no difference in age between MODS clusters in the present study. Shock on admission was only associated with the subsequent development of prolonged MODS found in cluster 3. There was a weaker association with crystalloid administration in this group than in cluster 1, and there was no association with the volume of blood product administration. These results suggest that the persistent MODS in patients in cluster 3 may be due to an early response to the shocked state, and not a later consequence of resuscitation. Prolonged MODS may represent the contemporary form of classical multiple organ failure, driven by dysregulation of the inflammatory response to injury^41^. Developing biomarkers and stratification tools to identify these patients early in the clinical course may allow the development of new management strategies and therapeutic opportunities for MODS after injury.

There are a number of limitations to this study. Although wide ranging geographically, it was limited in its accrual period and the number of data points obtained for each patient. In particular, it was not possible to capture the number and nature of infectious episodes or other complications that may have contributed to prolonged MODS. It was not possible to assign cause of death, which would have provided insight into whether and how MODS contributed to the death of each patient. In the hierarchical clustering analysis, selection of the number of clusters to analyse was based on determination of adequate cluster size for intergroup analyses. Further subtypes may be identified from deeper analysis of larger cohorts of patients. Although the majority of deaths in the early resolving MODS group occurred within 48 h, the patterns of death for the different clusters require further evaluation. SOFA scoring differs from other MODS scores (such as the Denver^42^ or Goris^43^ score) as it includes CNS and coagulation components, which may lead to differences in reported incidence of MODS^33^, and may partly account for differences in proportion of MODS in the present analysis compared with other studies. However, the use of SOFA in severely injured and TBI populations has good discriminative ability, and balance of sensitivity and specificity in predicting unfavourable outcome for injured patients^14^, ^44^. In the present multivariable analyses examining associations between clinical variables and the development of MODS, there was potential overlap between SOFA components as both admission (input) and later SOFA (output) variables, such as the Glasgow Coma Scale (GCS). GCS was not associated with the development of MODS, whereas the anatomical presence of a head injury was associated with MODS development only in cluster 2. The authors therefore believe that the findings provide a robust signal for the associations across each cluster analysis. In this observational study, however, it was only possible to identify associations between clinical variables and MODS; causation could not be determined. There were only 24 patients with MODS in cluster 3, which may have led to model overfit. Larger cohort studies are required to explore these smaller cohorts, and potential further subcluster divisions. Finally, the multicentre nature of this study may mean that treatment policies differed despite national guidance, so this study represents clinically focused descriptors of MODS subtypes rather than ones driven by biology alone.

MODS is still common in the era of damage control resuscitation, and has a high associated mortality rate and resource use. Contemporary MODS is now almost always present from admission, commonly involves cardiovascular dysfunction, and has subtypes with different patterns of recovery. Patients who develop persistent MODS are difficult to identify on admission based on their clinical characteristics. The development of tools for the early identification of these subtypes requires further research on their individual initiating mechanisms.

## Collaborators

ORDIT study collaborators: H. Akkad (Centre for Trauma Sciences, Queen Mary University of London, London, UK); K. Apostolidou (Cambridge University Hospitals NHS Foundation Trust, Cambridge, UK); R. Ardley (Nottingham University Hospitals NHS Trust, Nottingham, UK); C. Aylwin (Imperial College Healthcare NHS Trust, London, UK); C. Bassford (University Hospitals Coventry and Warwickshire NHS Trust, Coventry, UK); S. Bonner (South Tees Hospitals NHS Foundation Trust, Middlesbrough, UK); A. Brooks (Nottingham University Hospitals NHS Trust, Nottingham, UK); T. Cairns (Newcastle upon Tyne Hospitals NHS Foundation Trust, Newcastle upon Tyne, UK); M. Cecconi (St George's University Hospitals NHS Foundation Trust, London, UK); F. Clark (University Hospitals of North Midlands NHS Trust, Stoke‐on‐Trent, UK); G. Dempsey (Aintree University Hospitals NHS Foundation Trust, Liverpool, UK); E. Denison Davies (Lancashire Teaching Hospitals NHS Foundation Trust, Preston, UK); R. Docking (Queen Elizabeth University Hospital Glasgow, Glasgow, UK); J. Eddlestone (Central Manchester University Hospitals NHS Foundation Trust, Manchester, UK); D. Ellis (Central Manchester University Hospitals NHS Foundation Trust, Manchester, UK); J. Evans (Salford Royal NHS Foundation Trust, Salford, UK); M. Galea (University Hospital Southampton NHS Foundation Trust, Southampton, UK); M. Healy (Barts Health NHS Trust, London, UK); D. Horner (Salford Royal NHS Foundation Trust, Salford, UK); R. Howarth (Lancashire Teaching Hospitals NHS Foundation Trust, Preston, UK); J. Jansen (Aberdeen Royal Infirmary, Aberdeen, UK, and University of Alabama at Birmingham, Birmingham, Alabama, USA); J. Jones (Leeds Teaching Hospitals NHS Trust, Leeds, UK); C. Kaye (Aberdeen Royal Infirmary, Aberdeen, UK); J. Keep (King's College Hospital NHS Foundation Trust, London, UK); D. Kerslake (Royal Infirmary of Edinburgh, Edinburgh, UK); J. Kilic (Brighton and Sussex University Hospitals NHS Trust, Brighton, UK); M. Leong (Oxford University Hospitals NHS Foundation Trust, Oxford, UK); V. Martinson (Hull and East Yorkshire Hospitals NHS Trust, Hull, UK); B. McIldowie (North Bristol NHS Trust, Bristol, UK); S. Michael (Sheffield Teaching Hospitals NHS Foundation Trust, Sheffield, UK); J. Millo (Oxford University Hospitals NHS Foundation Trust, Oxford, UK); M. Morgan (University Hospital of Wales, Cardiff, UK); R. O'Leary (Cambridge University Hospitals NHS Foundation Trust, Cambridge, UK); J. Oram (Leeds Teaching Hospitals NHS Trust, Leeds, UK); L. Ortiz‐Ruiz De Gordoa (Brighton and Sussex University Hospitals NHS Trust, Brighton, UK); K. Porter (University Hospital Birmingham NHS Foundation Trust, Birmingham, UK); S. Raby (Oxford University Hospitals NHS Foundation Trust, Oxford, UK); J. Service (Queen Elizabeth University Hospital Glasgow, Glasgow, UK); D. Shaw (Royal Liverpool and Broadgreen University Hospitals NHS Trust, Liverpool, UK); J. D. Smith (Ninewells Hospital, Dundee, UK); N. Smith (Hull and East Yorkshire Hospitals NHS Trust, Hull, UK); M. Stotz (Imperial College Healthcare NHS Trust, London, UK); E. Thomas (Plymouth Hospitals NHS Trust, Plymouth, UK); M. Thomas (North Bristol NHS Trust, Bristol, UK); A. Vincent (Newcastle upon Tyne Hospitals NHS Foundation Trust, Newcastle upon Tyne, UK); G. Ward (University Hospitals Coventry and Warwickshire NHS Trust, Coventry, UK); I. Welters (Royal Liverpool and Broadgreen University Hospitals NHS Trust, Liverpool, UK).

## Supporting information


**Table S1** ADMISSION CHARACTERISTICS AND OUTCOMES FOR PATIENTS WITH AND WITHOUT TBI
**Table S2** CHARACTERISTICS OF PATIENTS WITH MODS PER SITE
**Table S3** ADMISSION CHARACTERISTICS AND OUTCOMES FOR PATIENTS WITH MODS IN QUARTILES OF CRYSTALLOID
**Fig. S1** Organ SOFA scores in all patients. Line graphs show mean (95% CI) individual organ component SOFA scores for a. Respiratory, b. Cardiovascular, c. Central Nervous, d. Coagulation, e. Hepatic and f. Renal systems by day of hospital admission in patients within Cluster 1 (C1) No MODS, C1 MODS, Cluster 2 (C2) MODS and Cluster 3 (C3) MODS
**Fig. S2** Organ SOFA scores in patients without TBI. Line graphs show mean (95% CI) individual organ component SOFA scores for a. Respiratory, b. Cardiovascular, c. Central Nervous, d. Coagulation, e. Hepatic and f. Renal systems by day of hospital admission in patients within C1 No MODS, C1 MODS, C2 MODS and C3 MODS
