# Supplementary material for: Multiple organ dysfunction after trauma
Source: Br J Surg. 2019 Nov 6;107(4):402–12. doi: 10.1002/bjs.11361 (PMC7078999; doi:10.1002/bjs.11361)

**BJS11361**

**Multiple organ dysfunction after trauma**

E. Cole, S. Gillespie, P. Vulliamy and K. Brohi, on behalf of the Organ Dysfunction in Trauma (ORDIT) study collaborators

**Table S1 ADMISSION CHARACTERISTICS AND OUTCOMES FOR PATIENTS WITH AND WITHOUT TBI**

|  | **No TBI No MODS** | **No TBI MODS** | **TBI No MODS** | **TBI MODS** |
| --- | --- | --- | --- | --- |
|  | ***164*** | ***146*** | ***31*** | ***99*** |
| Age | 48 (27-62) | 47 (30-59) | 38 (24-63) | 46 (28-65) |
| Male (%) | 114 (70) | 111 (76) | 25 (81) | 74 (75) |
| Blunt (%) | 128 (78) | 130 (89) | 30 (97) | 84 (85) |
| First GCS | 15 (9-15) | 14 (7-15)* | 13 (3-15) | 14 (6-15) |
| First SBP | 121 (115-129) | 119 (112-120) | 132 (110-152) | 127 (106-150) |
| Admission BD (mEq/L) | 0 (-1.3-0.8) | 0 (-0.8-6.4)** | 0 (-1.3-4.2) | 0.3 (-0.3-5.7) |
| CSL L/24H (L) | 0.5 (0.2-1.3) | 2.6 (1.5-3.8)** | 0.6 (0.3-1) | 2.8 (2.3-3.2)** |
| RBC units/24H | 3 (2-5) | 4 (2-8)* | 1 (0-3) | 3 (2-5)** |
| ISS | 16 (9-26) | 25 (13-30)** | 24 (15-31) | 29 (24-36)** |
| APACHE II score | 9 (6-12) | 14 (10-16)** | 13 (7-16) | 16 (10-23)* |
| ***Outcomes:*** |  |  |  |  |
| Mortality (%) | 1 (<1) | 22 (15)** | 0 | 32 (32)** |
| Ventilator days | 1 (0-1) | 3 (0-9)** | 0 (0-2) | 5 (2-11)** |
| CCLOS | 3 (2-4) | 9 (4-18)** | 3 (2-5) | 13 (6-21)** |
| THLOS | 9 (5-20) | 22 (12-39)** | 11 (4-23) | 29 (20-44)** |

Median (IQR) unless otherwise specified. *p<0.050; **p<0.010 when comparing No MODS and MODS groups without and with TBI. Abbreviations = GCS: Glasgow Coma Scale; SBP: Systolic Blood Pressure; BD: Base Deficit; CSL: Crystalloid; RBC: Red Blood Cells; TBI: Traumatic Brain Injury; ISS: Injury Severity Score; APACHE II: Acute Physiology and Chronic Health Evaluation II; CCLOS: Critical Care Length of stay; THLOS: Total Hospital Length of stay.

**Table S2 CHARACTERISTICS OF PATIENTS WITH MODS PER SITE**

| **Site** | **N enrol** | **% MODS** | **% TBI** | **Age** | **First GCS** | **First SBP** | **ISS** | **APACHE II** | **N (%) mortality** |
| --- | --- | --- | --- | --- | --- | --- | --- | --- | --- |
| 1 | 34 | 56% | 32% | 54 (21-67) | 7 (4-13) | 124 (92-126) | 29 (16-43) | 11 (9-14) | 2 (11) |
| 3 | 9 | 78% | 14% | 49 (47-62) | 13 (9-15) | 115 (112-153) | 29 (16-34) | 19 (16-22) | 0 |
| 4 | 30 | 40% | 17% | 63 (56-72) | 13 (4-15) | 127 (112-136) | 36 (27-45) | 13 (8-24) | 6 (50) |
| 5 | 6 | 50% | 0% | 61 (51-86) | 8 (3-15) | 129 (102-163) | 14 (1-20) | 14 (6-26) | 1 (33) |
| 6 | 7 | 100% | 57% | 41 (26-50) | 9 (5-14) | 114 (95-144) | 18 (8-38) | 26 (22-28) | 0 |
| 7 | 11 | 64% | 45% | 55 (24-66) | 9 (4-15) | 130 (113-152) | 38 (25-57) | 17 (4-19) | 1 (9) |
| 8 | 10 | 50% | 0% | 46 (31-66) | 13 (8-15) | 100 (53-134) | 29 (12-35) | 11 (9-19) | 0 |
| 9 | 19 | 42% | 25% | 39 (24-52) | 12 (3-15) | 106 (72-146) | 39 (25-55) | 15 (8-16) | 1 (13) |
| 10 | 10 | 30% | 0% | 52 (51-87) | 15 (4-15) | 122 (98-160) | 16 (9-30) | 11 (9-13) | 2 (67) |
| 11 | 14 | 93% | 46% | 30 (23-66) | 7 (3-14) | 123 (107-135) | 43 (32-46) | 10 (7-14) | 2 (15) |
| 12 | 19 | 16% | 0% | 56 (26-74) | 8 (7-14) | 121 (108-153) | 27 (16-38) | 10 (6-28) | 2 (33) |
| 13 | 16 | 81% | 0% | 56 (43-72) | 11 (7-15) | 126 (103-133) | 25 (18-39) | 19 (16-22) | 2 (15) |
| 14 | 3 | 67% | 0% | 55 (36-73) | 14 (14-15) | 134 (130-137) | 10 (9-10) | 12 (6-18) | 0 |
| 15 | 39 | 74% | 59% | 37 (26-63) | 9 (6-15) | 122 (104-152) | 29 (25-34) | 11 (9-15) | 4 (10) |
| 16 | 6 | 83% | 0% | 34 (28-48) | 14 (10-15) | 110 (95-139) | 9 (9-25) | 8 (5-19) | 0 |
| 17 | 12 | 8% | 0% | 18 | 15 | 88 | 29 | 12 | 0 |
| 18 | 14 | 43% | 33% | 46 (41-77) | 8 (3-15) | 132 (128-142) | 18 (8-28) | 14 (10-26) | 2 (33) |
| 19 | 24 | 46% | 54% | 64 (39-72) | 11 (8-14) | 130 (113-169) | 23 (14-26) | 16 (10-24) | 5 (21) |
| 20 | 17 | 53% | 78% | 41 (29-50) | 4 (3-10) | 137 (80-154) | 34 (26-45) | 10 (7-18) | 2 (22) |
| 21 | 36 | 53% | 21% | 50 (32-79) | 6 (3-15) | 129 (105-140) | 19 (10-29) | 17 (13-18) | 3 (16) |
| 22 | 17 | 94% | 47% | 55 (33-70) | 13 (3-15) | 119 (84-140) | 25 (12-34) | 19 (14-25) | 4 (24) |
| 23 | 18 | 67% | 64% | 49 (33-69) | 10 (3-15) | 145 (112-156) | 25 (21-32) | 14 (10-17) | 5 (28) |
| 24 | 13 | 69% | 56% | 68 (48-80) | 14 (7-15) | 137 (119-165) | 25 (19-27) | 15 (12-20) | 2 (22) |
| 25 | 3 | 33% | 100% | 16 | 3 | 124 | 25 | 7 | 0 |
| 26 | 8 | 63% | 80% | 57 (34-67) | 12 (4-14) | 159 (113-179) | 9 (4-34) | 22 (16-29) | 2 (40) |
| 27 | 33 | 36% | 58% | 50 (41-57) | 15 (12-15) | 121 (103-140) | 14 (9-21) | 13 (9-16) | 3 (25) |
| 28 | 2 | 100% | 100% | 29 (17-40) | 8 (8-8) | 126 (143-149) | 29 (20-38) | 20 (19-21) | 0 |
| 29 | 9 | 89% | 50% | 55 (39-66) | 10 (5-15) | 123 (72-172) | 18 (12-58) | 17 (12-22) | 3 (38) |
| Average | 15.6 | 59% | 52% | 48 (30-62) ᶧ | 10 (4-14) ᶧ | 124 (104-123) ᶧ | 24 (16-34) ᶧ | 14 (10-20) ᶧ | 2 (23) |

**Data presented for patients who developed MODS**. ᶧ Median (IQR). Abbreviations = TBI: Traumatic Brain Injury; GCS: Glasgow Coma Scale; SBP: Systolic Blood Pressure; ISS: Injury Severity Score; APACHE II: Acute Physiology and Chronic Health Evaluation II. ORDIT site 2 enrolled two patients, neither of which developed MODS and sites 17 and 25 only had one patient who developed MODS.

**Table S3 ADMISSION CHARACTERISTICS AND OUTCOMES FOR PATIENTS WITH MODS IN QUARTILES OF CRYSTALLOID**

| **Crystalloid Volume** | **<1.5L** | **1.5 - 2.6L** | **2.6 - 3.9L** | **>3.9L** |
| --- | --- | --- | --- | --- |
|  | ***57*** | ***64*** | ***52*** | ***72*** |
| Age | 45 (31-65) | 49 (30-62) | 48 (32-68) | 46 (28-57) |
| Male (%) | 42 (74) | 47 (73) | 42 (81) | 50 (69) |
| Blunt (%) | 50 (88) | 60 (94) | 48 (92) | 62 (85) |
| First GCS | 13 (5-15) | 14 (6-15) | 14 (5-14) | 14 (7-15) |
| First SBP | 127 (110-145) | 123 (94-137) | 127 (104-142) | 126 (103-149) |
| Admission BD (mEq/L) | 0 (-0.1-4.0) | 0 (-0.4-6.9) | 0.05 (-0.5-6.8) | 2.1 (0.1-7.0) |
| CSL L/24H (L) | 0.25 (0-1) | 2 (1.7-2.2) | 2.9 (2.6-3.3) | 5.5 (4.9-6.0)** |
| RBC units/24H | 4 (1-6) | 5 (2-7) | 4 (2-6) | 5 (2-6) |
| TBI (%) | 26 (46) | 25 (39) | 21 (40) | 27 (37) |
| ISS | 25 (13-30) | 26 (17-33) | 25 (18-38) | 26 (19-32) |
| APACHE II score | 17 (10-18) | 15 (11-22) | 14 (10-18) | 13 (9-21) |
| ***Outcomes:*** |  |  |  |  |
| Mortality (%) | 9 (16) | 21 (33) | 10 (19) | 14 (19) |
| Ventilator days | 3 (1-8) | 3 (1-11) | 4 (1-11) | 5 (2-12) |
| CCLOS | 6 (3-12) | 7 (4-16) | 10 (4-20) | 13 (6-20)** |
| THLOS | 23 (12-40) | 25 (10-40) | 25 (13-35) | 33 (18-47) |

Median (IQR) unless otherwise specified. **p<0.010 when comparing four groups, no comparisons reached p<0.05. Abbreviations = GCS: Glasgow Coma Scale; SBP: Systolic Blood Pressure; BD: Base Deficit; CSL: Crystalloid; RBC: Red Blood Cells; TBI: Traumatic Brain Injury; ISS: Injury Severity Score; APACHE II: Acute Physiology and Chronic Health Evaluation II; CCLOS: Critical Care Length of stay; THLOS: Total Hospital Length of stay.

**Fig. S1** Organ SOFA scores in all patients. Line graphs show mean (95% CI) individual organ component SOFA scores for a. Respiratory, b. Cardiovascular, c. Central Nervous, d. Coagulation, e. Hepatic and f. Renal systems by day of hospital admission in patients within Cluster 1 (C1) No MODS, C1 MODS, Cluster 2 (C2) MODS and Cluster 3 (C3) MODS
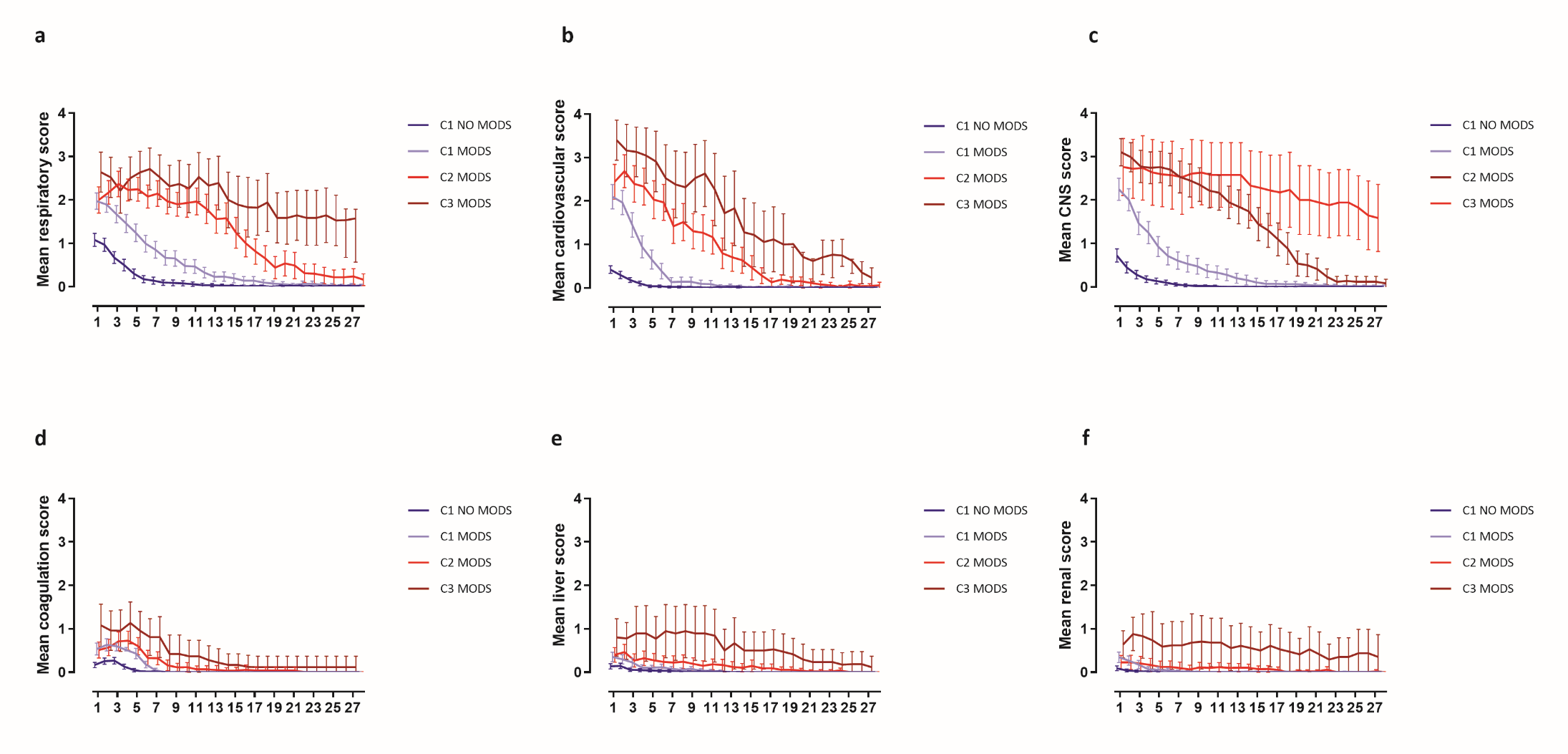


**Fig. S2** Organ SOFA scores in patients without TBI. Line graphs show mean (95% CI) individual organ component SOFA scores for a. Respiratory, b. Cardiovascular, c. Central Nervous, d. Coagulation, e. Hepatic and f. Renal systems by day of hospital admission in patients within C1 No MODS, C1 MODS, C2 MODS and C3 MODS
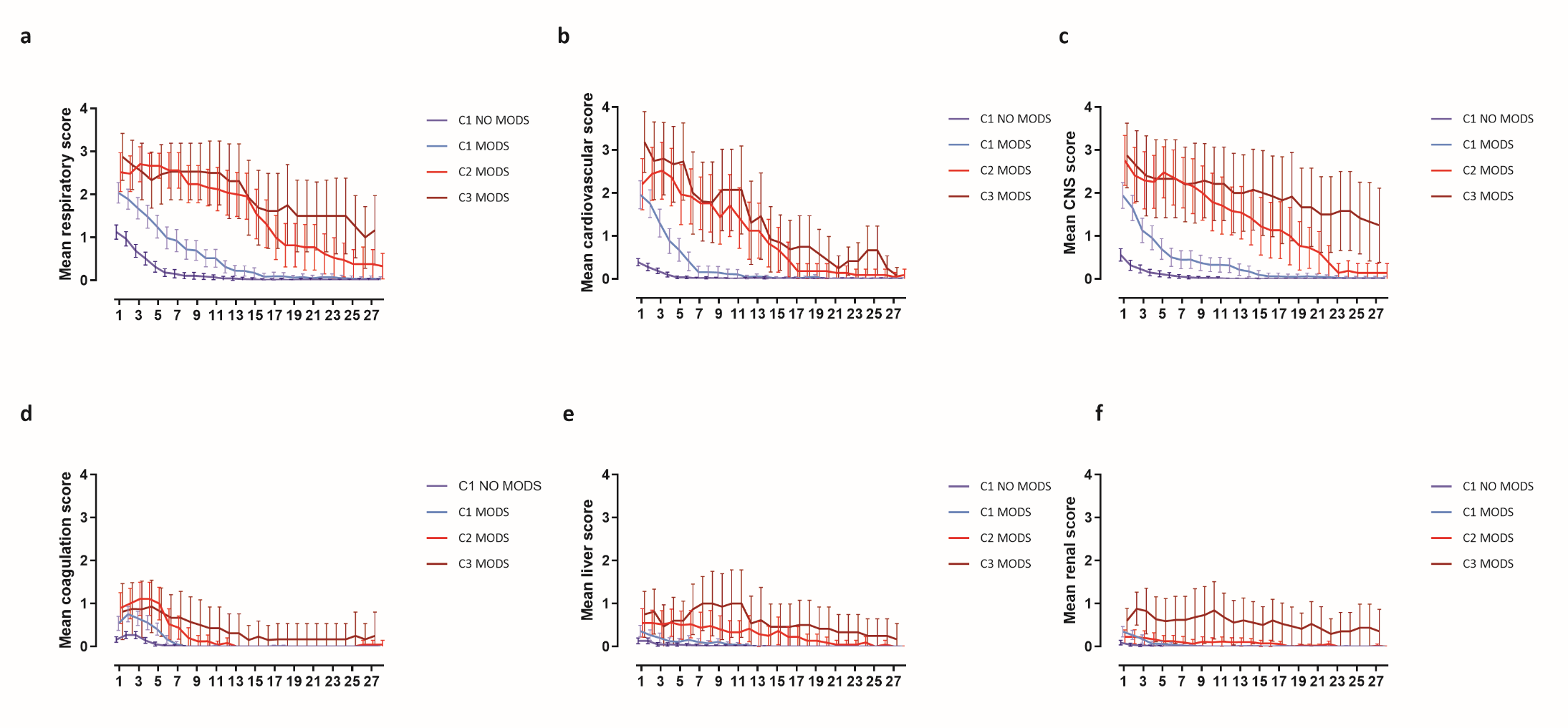

Supplement: Supplementary file 1 — Table S1 ADMISSION CHARACTERISTICS AND OUTCOMES FOR PATIENTS WITH AND WITHOUT TBI Table S2 CHARACTERISTICS OF PATIENTS WITH MODS PER SITE Table S3 ADMISSION CHARACTERISTICS AND OUTCOMES FOR PATIENTS WITH MODS IN QUARTILES OF CRYSTALLOID Fig. S1 Organ SOFA scores in all patients. Line graphs show mean (95% CI) individual organ component SOFA scores for a. Respiratory, b. Cardiovascular, c. Central Nervous, d. Coagulation, e. Hepatic and f. Renal systems by day of hospital admission in patients within Cluster 1 (C1) No MODS, C1 MODS, Cluster 2 (C2) MODS and Cluster 3 (C3) MODS Fig. S2 Organ SOFA scores in patients without TBI. Line graphs show mean (95% CI) individual organ component SOFA scores for a. Respiratory, b. Cardiovascular, c. Central Nervous, d. Coagulation, e. Hepatic and f. Renal systems by day of hospital admission in patients within C1 No MODS, C1 MODS, C2 MODS and C3 MODS [file BJS-107-402-s001.docx]
